# Galectin-1 promotes vasculogenic mimicry in gastric adenocarcinoma via the Hedgehog/GLI signaling pathway

**DOI:** 10.18632/aging.104000

**Published:** 2020-11-10

**Authors:** Xiaolan You, Jian Wu, Yuanjie Wang, Qinghong Liu, Zhiyi Cheng, Xiaojun Zhao, Guiyuan Liu, Chuanjiang Huang, Jiawen Dai, Yan Zhou, Dehu Chen, Yang Chong

**Affiliations:** 1 Department of Gastrointestinal Surgery, Hospital Affiliated 5 to Nantong University (Taizhou People’s Hospital) Taizhou, Jiangsu Province, China

**Keywords:** Galectin-1, vasculogenic mimicry, hedgehog/GLI signaling, gastric cancer

## Abstract

Background: Galectin-1 (GAL-1), which is encoded by *LGALS1*, promotes vasculogenic mimicry (VM) in gastric cancer (GC) tissue. However, the underlying mechanism remains unclear.

Methods: Immunohistochemical (IHC) and CD34-periodic acid-Schiff (PAS) double staining were used to investigate Glioma-associated oncogene-1(GLI1) expression and VM in paraffin-embedded sections from 127 patients with GC of all tumor stages. *LGALS1* or *GLI1* were stably transduced into MGC-803 cells and AGS cells, and western blotting, IHC, CD34-PAS double staining and three-dimensional culture *in vitro,* and tumorigenicity *in vivo* were used to explore the mechanisms of GAL-1/ GLI1 promotion of VM formation in GC tissues.

Results: A significant association between GAL-1 and GLI1 expression was identified by IHC staining, as well as a significant association between GLI1 expression and VM formation. Furthermore, overexpression of *LGALS1* enhanced expression of *GLI1* in MGC-803 and AGS cells. *GLI1* promoted VM formation both *in vitro* and *in vivo.* The effects of *GLI1* on VM formation were independent of *LGALS1*. Importantly, the expression of VM-related molecules, such as MMP2, MMP14 and laminin5γ2, was also affected upon *GLI1* overexpression or silencing in GC cell lines. Conclusion: GAL-1 promotes VM in GC through the Hh/GLI pathway, which has potential as a novel therapeutic target for treatment of VM in GC.

## INTRODUCTION

Cancer is a major public health problem worldwide and is the second leading cause of death in the United States [1]. In China, gastric cancer (GC) has high incidence and mortality rates, and the 5-year survival rate of advanced GC is <t;30% [2]. Previously, endothelium-dependent vessels were considered to be the sole blood source for gastric cancer tumors. The emergence of a vascular endothelial growth factor receptor (VEGFR)-2 antagonist monoclonal antibody holds potential as a therapy for patients with advanced GC, particularly patients for whom chemotherapy is not beneficial [3]. However, the therapeutic effect of the VEGFR-2 antagonist currently remains unsatisfactory [4], which may be due to nonendothelial vasculogenic mimicry (VM). Nonendothelial vasculature is comprised of highly aggressive and metastatic tumor cells, includes an internal basement membrane and a lack of endothelial cells in the internal lining. VM has been identified in multiple malignant tumors, including osteosarcoma [5], bladder cancer [6], breast cancer [7], prostate cancer [8], Glioma [9], ovarian Cancer [10], pancreatic cancer [11] and gastric adenocarcinoma [12]. Many studies have reported VM in a variety of poorly differentiated malignancies [7, 8, 12]. It has been reported that in the case of GC, VM not only provides a tumor blood supply, but also facilitates tumor metastases and VM of primary GC tissue. It is also an indicator of poor prognosis for GC patients after gastrectomy [13].

Galectins are a lectin family of 15 members that bind to carbohydrates, and contain one or multiple carbohydrate recognition domains. Recent studies have found that Galectin-1 (GAL-1) is highly expressed in a variety of malignant tumors, and that it is involved in various malignant functions, including tumorigenesis, development, invasion and metastasis, angiogenesis and immune escape [14]. However, its roles in VM remain largely uncharacterised. Our previous work demonstrated that GAL-1 can promote VM formation in GC by upregulating epithelial-mesenchymal transition (EMT) signaling [15], and that GAL-1 can induce EMT through upregulation of Hedgehog (Hh) signaling via Glioma-associated oncogene-1 (GLI1) in GC [16, 17]. The present study is based on the hypothesis that GAL-1 may also promote VM formation via GLI1 by activating the Hh signaling pathway in GC. In this study, we explored whether the GAL-1-Hh/ GLI signaling pathway was the underlying mechanism of VM in GC.

## RESULTS

### GLI1 is overexpressed in GC tissues

**Figure 1:**
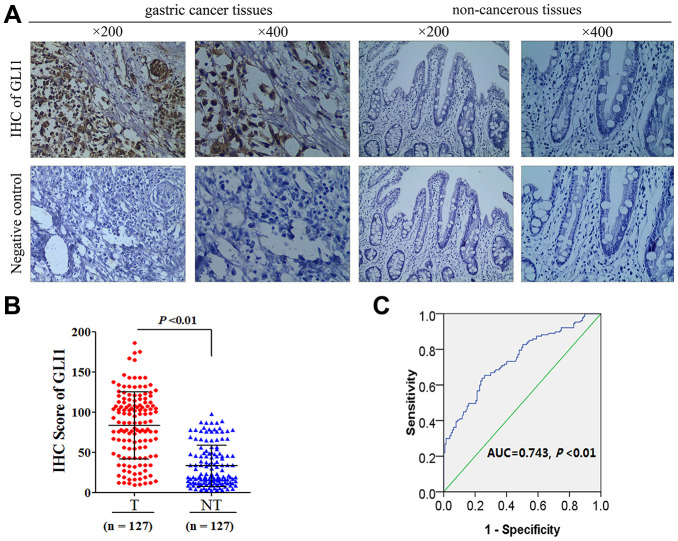
****GLI1 is overexpressed in GC tissues (**A**) Representative images of IHC for GLI1 protein expression in GC tissues and matched non-tumor tissues. (**B**) GLI1 IHC scoring compared between tumor and matched non-tumor tissues. (**C**) ROC statistics were employed to estimate the cut-off points of GLI1 IHC scoring in human GC tissue.

In order to understand the role of Hh signaling in VM in GC, we detected the expression of GLI1 in 127 GC tissues and matched non-tumor tissues. For the GC tissues, the median GLI1 IHC score was 65.08 (6.73-164.78), whereas that of the matched non-tumor tissues was 35.21 (3.16-98.12) (Figure 1A). A significant difference in GLI1 expression was observed between GC and non-tumor tissues (*P* < 0.01; Figure 1B). The receiver operating curve statistic was used to distinguish positive and negative expression of GLI1 in 127 GC samples in accordance with the cutoff point for IHC scores. Scores ≥ 78.38 were considered to indicate positive expression (Figure 1C). This indicated a rate of positive GLI1 expression in GC tissues of 39.4% (50/127), and 8.66% (11/127) in the matched non-tumor tissues, which is a significant difference (χ^2^ = 32.81; *P* < 0.01).

### GAL-1 expression is correlated with GLI1, and GLI1 expression is correlated with VM

**Figure 2:**
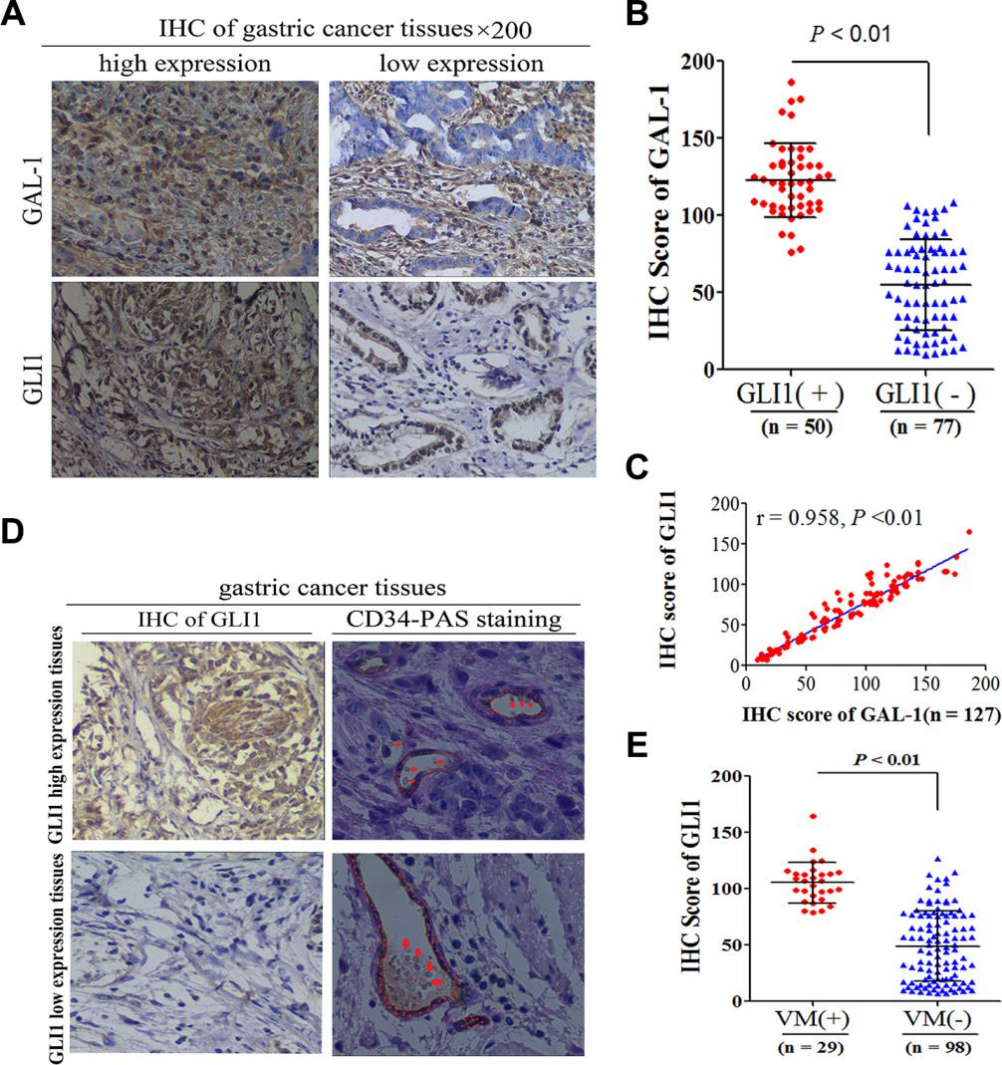
**GAL-1 expression is correlated with GLI1, and GLI1 expression is correlated with VM.** (**A**) Representative images of IHC indicating GLI1 and GAL-1 protein expression in GC tissues (×200 magnification). (**B**) The GAL-1 IHC scores in GLI1-positive GC tissues were significantly higher than the GAL-1 IHC scores in GLI1-negative GC tissues(*P* < 0.01) (**C**) GAL-1 IHC scores were positively correlated with GLI1 IHC scores in GC tissues (r = 0.958; *P* < 0.01). (**D**) GC tissues exhibiting high GLI1 expression were more likely to form VM (endogenous cell-dependent vessels are indicated by the red dotted line, VM is indicated by the green dotted line, and red blood cells are indicated by red arrows in VM and endogenous cell-dependent vessels; ×400 magnification). (**E**) The GLI1 IHC scores in GC tissues with VM were significantly higher than those without VM (*P* < 0.01).

To determine whether GAL-1 can activate Hh signaling and induce VM in GC tissue, we examined GAL-1 in GC tissue using IHC and examined VM using CD34/PAS double-staining. The median IHC score for GAL-1was 78.29 (9.51-186.24) in GC tissues. The GAL-1 IHC scores in GLI1-positive GC tissues were significantly higher than the GAL-1 IHC scores in GLI1-negative GC tissues (*P* <0.01; Figure 2A, 2B). GAL-1 IHC scores positively correlate with GLI1 IHC scores in GC tissues (r = 0.958; *P* < 0.01; Figure 2C). As shown in Figure 2D, one GC specimen exhibited endogenous cell-dependent vessels and VM. Red blood cells are indicated by the red arrow in VM and endogenous cell-dependent vessels. CD34/PAS double staining indicated VM in 29/127 cases (22.8%). GC tissues with GLI1 high expression were more likely to exhibit VM. The GLI1 IHC scores of GC tissues with VM were significantly higher than those without VM (*P* < 0.01; Figure 2E). These results suggested that GAL-1 may contribute to VM formation in GC through activating Hh signaling and promoting GLI1 expression.

### GAL-1 promotes GLI1 expression both *in vitro* and *in vivo*

**Figure 3:**
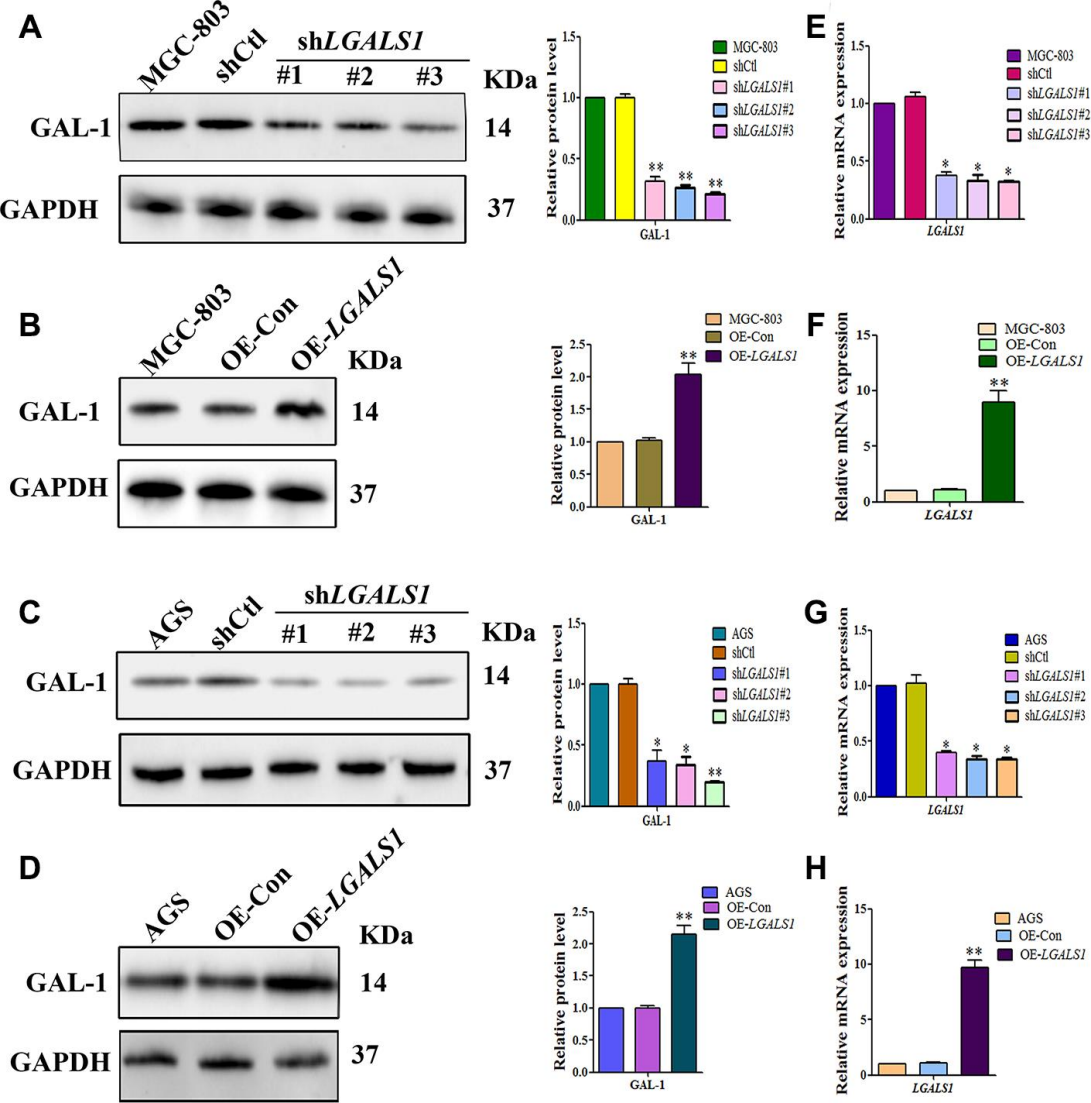
**Lentiviral vectors were used to overexpress or silencing *LGALS1* using 3 shRNAs.** Western blot analysis showing decreased GAL-1 protein expression in (**A**) MGC-803 cells, and (**C**) AGS cells transfected with *LGALS1* shRNAs, compared with cells transfected with control shRNA (shCtl). Western blot showing GAL-1 protein expression following overexpression of *LGALS1* (OE-*LGALS1*) compared to the empty vector (OE-Con) in (**B**) MGC-803 cells, and (**D**) AGS cells. Reduced expression levels of *LGALS1* mRNA in (**E**) MGC-803 cells, and (**G**) AGS cells transfected with *LGALS1* shRNAs. Increased expression levels of *LGALS1* with OE*-LGALS1* in (**F**) MGC-803 cells, and (**H**) AGS cells transfected with OE-*LGALS1*. GAPDH was used as a loading control. (All n = 3, ^*^P < 0.05, ^**^P < 0.01).

**Figure 4:**
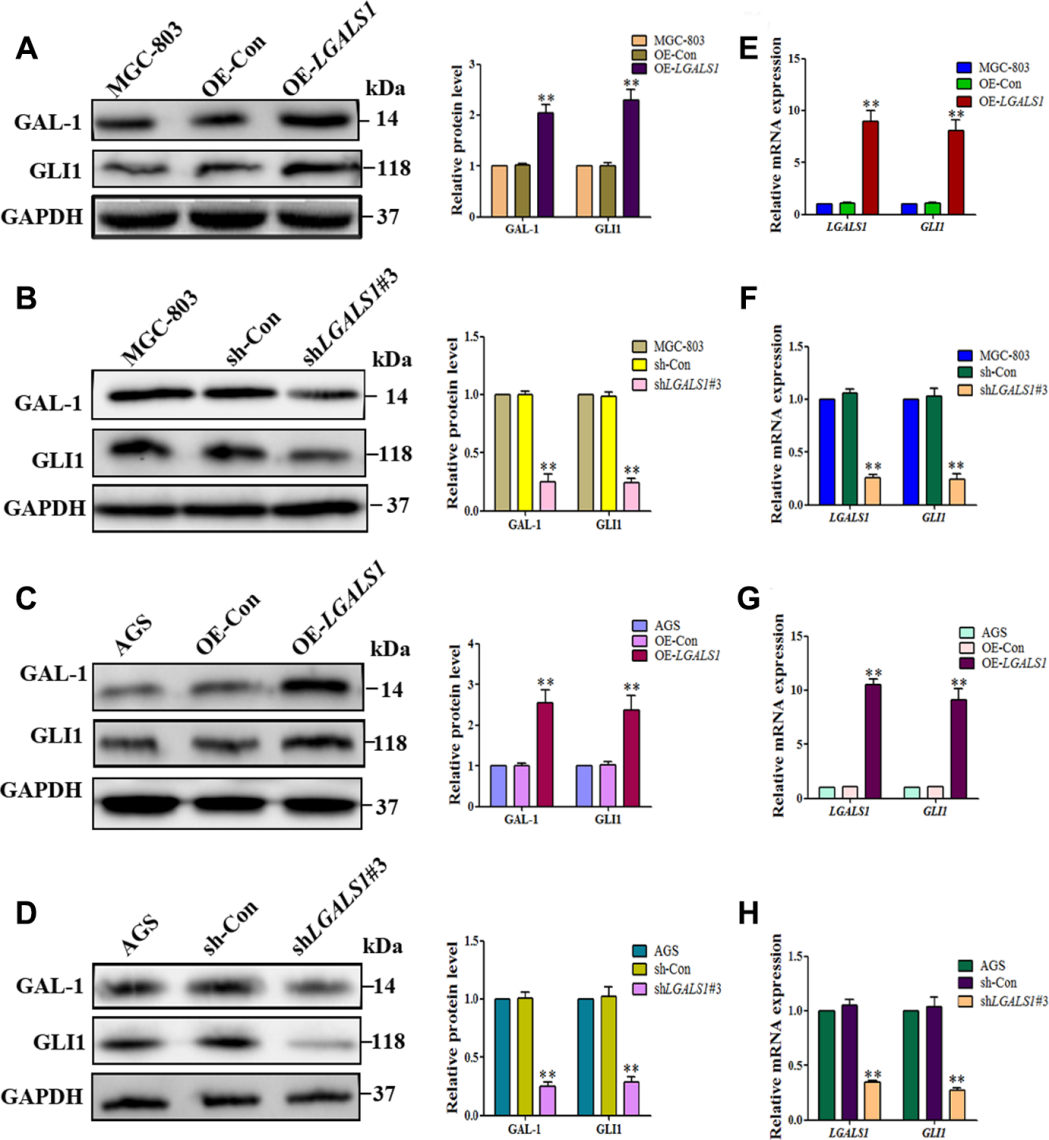
***L*GALS1 promotes GLI1 expression *in vitro*.** (**A** and **C**) Western blotting indicating that OE- *LGALS1* efficiently induced GLI1 protein expression compared with wild-type control and negative control-transduced cells. (**B** and **D**) Transfection with sh*LGALS1*#3 inhibited GLI1 protein expression compared with wild-type control and negative control-transduced cells. These results were confirmed at the mRNA level by qRT-PCR (**E**–**H**). GAPDH served as a loading control. (All n = 3, ^*^P < 0.05, ^**^P < 0.01).

To confirm whether GAL-1 activation of Hh signaling promotes GLI1 expression in GC, *LGALS1* was overexpressed and silencing in MGC-803 and AGS cells, confirmed by western blotting (Figure 3A–3D) and qRT-PCR (Figure 3E–3H). According to these results, shGal1#3 and LV-*LGALS1*-OE were selected for silencing and overexpression of GAL-1 in subsequent experiments, respectively. Western blotting indicated that *LGALS1*-overexpressing (OE-*LGALS1*) MGC-803 cells efficiently induced GLI1 protein expression compared with untransduced MGC-803 cells (wild-type control (WC) or negative control-transfected (OE-CON) cells) (Figure 4A). *LGALS1*- silencing (sh-*LGALS1*) MGC-803 cells efficiently inhibited GLI1 protein expression compared with WC or sh-CON MGC-803 cells (Figure 4B). We next performed qRT-PCR to evaluate *LGALS1* and *GLI1* mRNA expression in all cell lines, the results of which were consistent with those of western blotting (Figure 4E and 4F).

To confirm that GAL-1 promotes GLI1 expression *in vitro*, we repeated these experiments in AGS cells. OE-*LGALS1* AGS cells efficiently induced GLI1 protein expression (Figure 4C) and sh-*LGALS1* AGS cells efficiently inhibited GLI1 protein expression (Figure 4D). These results were confirmed at the mRNA level by qRT-PCR (Figure 4G and 4H).

**Figure 5:**
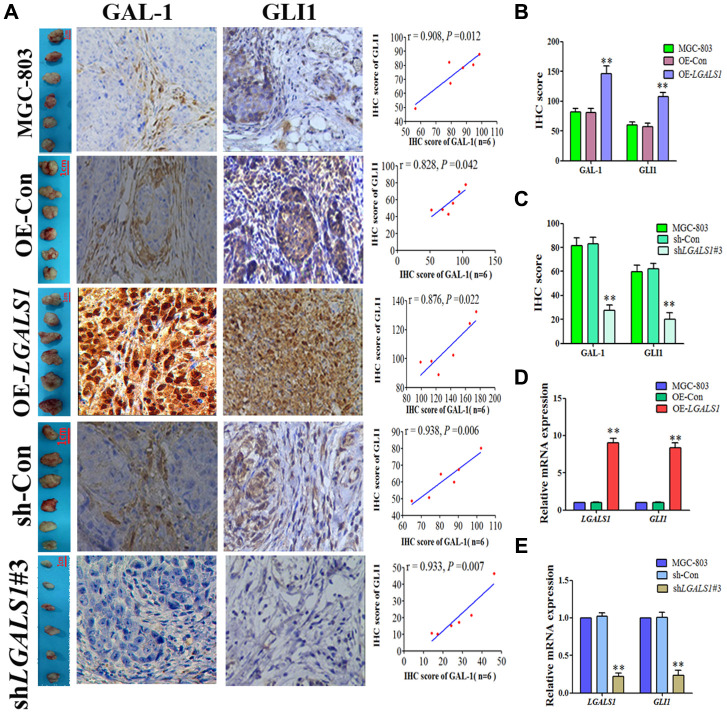
**LGALS1 enhances GLI1 expression *in vivo*.** (**A**) GLI1 and GAL-1 IHC scores were positively correlated in subcutaneous GC tissues from all groups (*P* < 0.05; ×200 magnification). (**B**) IHC scores indicating that GLI1 expression was elevated in the OE-*LGALS1* group (*P* < 0.01). (**C**) IHC scores indicating that GLI1 expression was abrogated in the sh*LGALS1*#3 group (*P* < 0.01). *LGALS1* and *GLI1* mRNA expression levels in (**D**) OE *LGALS1*-transduced and untransduced cells, and (**E**) shLGALS1#3-transduced and untransduced cells.

IHC scores of GC tissues from the subcutaneous GC mice indicated that GLI1 expression was positively correlated with that of GAL-1 in all groups (*P* < 0.05; Figure 5A). Compared with the WC and OE-CON groups, GLI1 expression was elevated in the OE-*LGALS1* group (*P* < 0.01; Figure 5B) and reduced in the sh-*LGALS1* group (*P* < 0.01; Figure 5C). *LGALS1* and *GLI1* mRNA expression was consistent with the IHC results (Figure 5D and 5E).

### GLI1 promotes VM *in vivo* and *in vitro*

**Figure 6:**
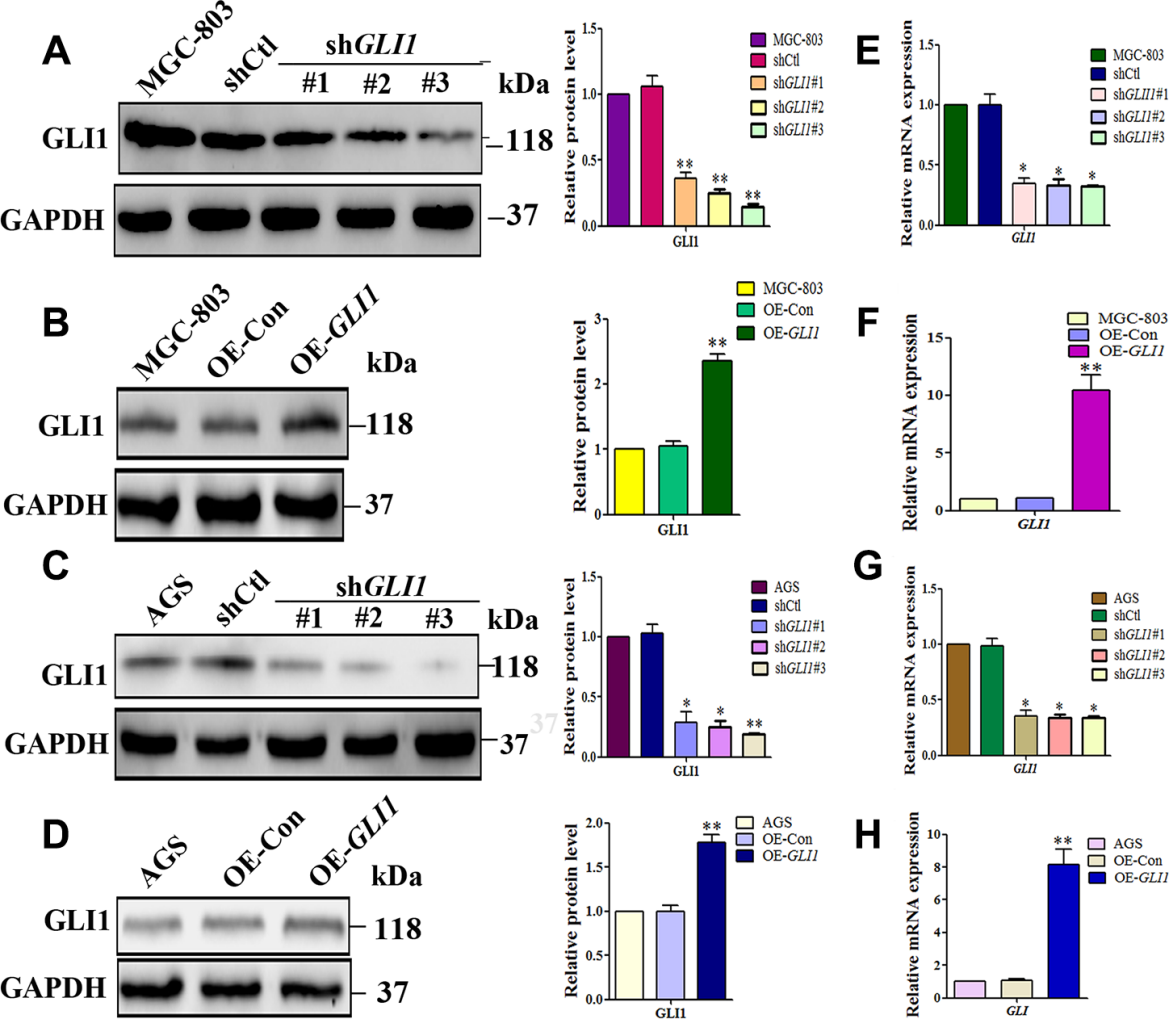
**Lentiviral vectors were used to overexpress or silencing *GLI1*.** Western blot analysis of GLI1 protein expression in (**A**) MGC-803 cells, and (**C**) AGS cells transfected with control shRNA (shCtl) or different *GLI1* targeting sequences (sh*GLI1*#1, sh*GLI1*#2, sh*GLI1*#3). Western blot showing GLI1 protein expression following overexpression of *GLI1* (OE-*GLI1*) or the empty vector (OE-CON) transfection in (**B**) MGC-803 cells, and (**D**) AGS cells. Expression levels of *GLI1* mRNA in (**E** and **G**) sh*GLI1* transduced and untransduced cells, and (**F** and **H**) OE-*GLI1* transduced and untransduced cells. GAPDH served as a loading control. (n = 3, ^*^P < 0.05, ^**^P < 0.01).

To confirm whether *GLI1* promotes VM *in vivo* and *in vitro*, lentiviral vectors were used to silencing or overexpress *GLI1* in MGC-803 and AGS cells. *GLI1* silencing and overexpression was confirmed by western blotting (Figure 6A–6D) and qRT-PCR (Figure 6E–6H). According to these results, sh*GLI1*#3 (for *GLI1* silencing) and LV-*GLI1*-OE (for *GLI1* overexpression) were selected for subsequent experiments *in vivo* and *in vitro*.

**Figure 7:**
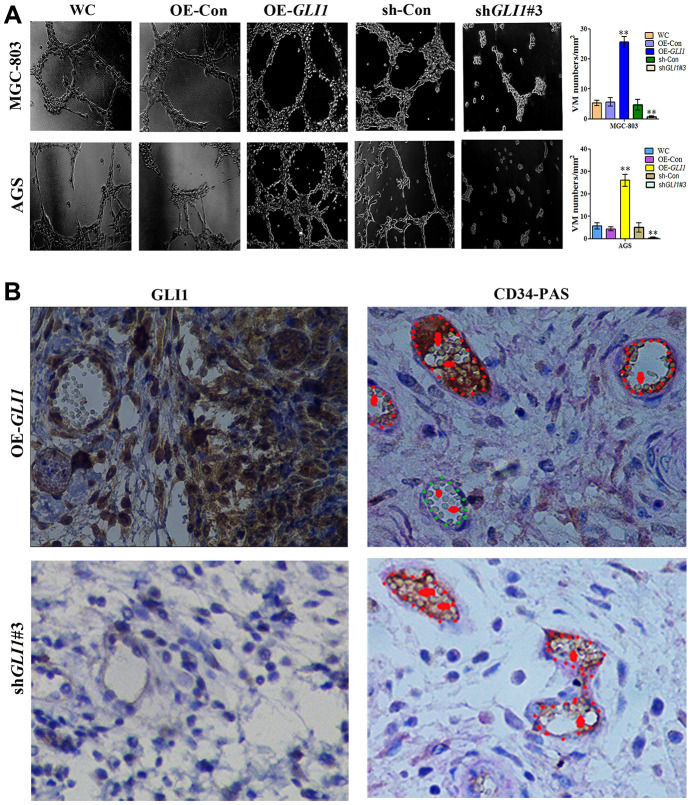
***GLI1* promotes VM *in vivo* and *in vitro*.** (**A**) Matrigel three-dimensional culture showing that *GLI1* overexpression enhanced tube formation in MGC-803 and AGS cells, while silencing of *GLI1* in MGC-803 and AGS cells inhibited their ability to form tube-like structures (×40 magnification; n = 3). (**B**) Expression of GLI1 and the formation of VM structures in the OE-*GLI1* and sh-*GLI1* subcutaneous GC mouse groups detected by IHC and CD34/PAS double staining. VM was significantly increased in the OE-*GLI1* group, and was absent in the sh*GLI1#3* group (endogenous cell-dependent vessels are indicated by the red dotted line, VM is indicated by the green dotted line, and red blood cells are indicated by red arrows in VM and endogenous cell-dependent vessels; ×400 magnification).

Matrigel three-dimensional culture showed that OE-*GLI1* transduction efficiently increased the capacity for tube-formation compared with WC or OE-CON MGC-803 and AGS cells. Meanwhile, sh-*GLI1* transduction reduced the ability to form tube-like structures compared with WC or sh-CON MGC-803 and AGS cells (Figure 7A). Following subcutaneous GC implantation, the expression of *GLI1* and the formation of VM structures in the OE-*GLI1* and sh-G*LI1* groups were detected by IHC and CD34/PAS double staining. CD34/PAS staining indicated that VM was significantly increased in the OE-*GLI1* group and was not observed in the tumor tissues from the sh-*GLI1* group (Figure 7B).

### GAL-1 promotes VM via the Hh signaling pathway

**Figure 8:**
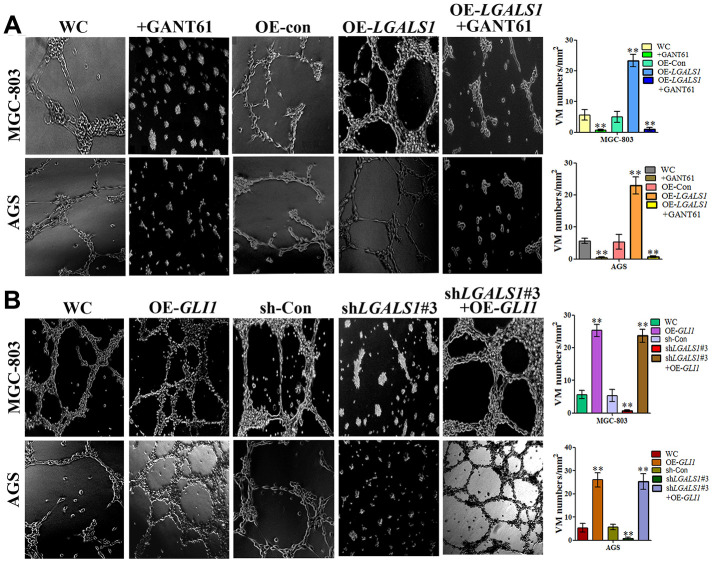
**GAL-1 increases VM through the Hh signaling pathway.** (**A**) Matrigel three-dimensional culture indicating that OE-*LGALS1* efficiently increased tube-formation by MGC-803 cells and AGS cells, and the inhibition of this effect by GANT61. (**B**) sh-*LGALS1* reduces the ability of MGC-803 cells and AGS cells to form tube-like structures. Simultaneous silencing of *LGALS1* and overexpression of *GLI1* rescued this reduction in tube-formation capacity. (×40 magnification; n=3).

To investigate whether GAL-1 promotes VM in GC through activation of the Hh pathway, the GLI1-specific antagonist, GANT61, was used to explore the relationship between GAL-1-induced VM in GC and Hh pathway activation. An MTT assay indicated that 10 μM GANT61 did not affect cell number at 24 h. However, Matrigel three-dimensional culture demonstrated that OE-*LGALS1* efficiently increased tube-formation capacity in MGC-803 cells and AGS cells, and that GANT61 can significantly inhibit this effect (Figure 8A). Meanwhile, sh-*LGALS1* reduced formation of tube-like structures in MGC-803 cells and AGS cells. However, simultaneous silencing of *LGALS1* and overexpression of *GLI1* in MGC-803 cells and AGS cells rescued this reduction in tube-formation capacity (Figure 8B). These results suggest that GLI1 affects VM formation independent of GAL-1.

**Figure 9:**
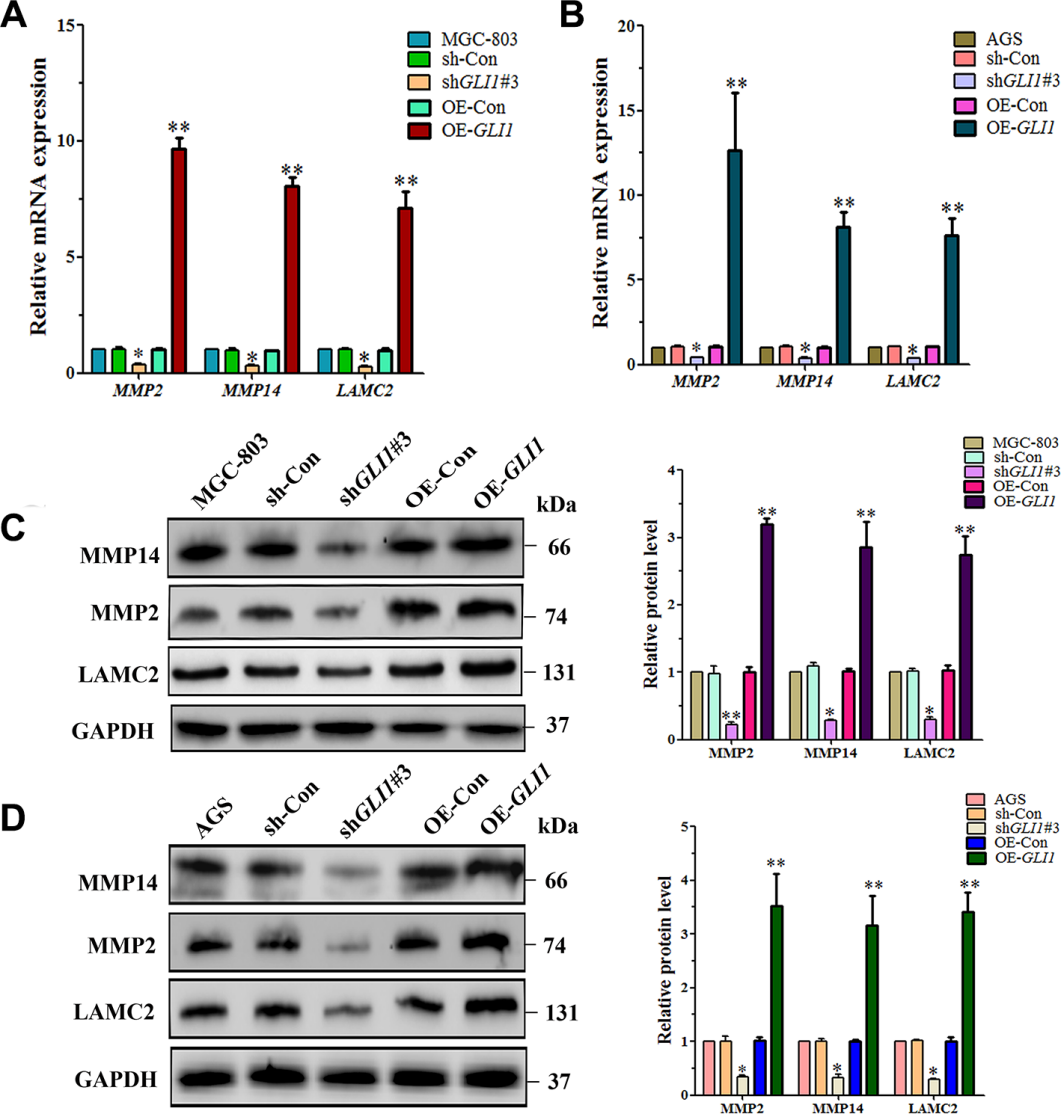
***GLI1* affects expression of VM markers - in MGC-803 cells and AGS cells.** qRT-PCR analysis of *MMP14, MMP2* and *LAMC2* mRNA expression in (**A**) MGC-803 cells, and (**B**) AGS cells. Western blot analysis of MMP14, MMP2, and LAMC2 protein expression in (**C**) MGC-803 cells, and (**D**) AGS cells. (All n=3, ^*^P < 0.05, ^**^P < 0.01).

To further investigate regulation of VM by *GLI1*, the expression of VM-related molecules was measured by qRT-PCR and western blotting in OE-*GLI1* and sh-G*LI1* MGC-803 and AGS cells, including MMP14, MMP2 and Ln5γ2 (LAMC2). We found that the mRNA expression of *MMP2* (*P* < 0.05), *MMP-14* (*P* < 0.05) and *LAMC2* (*P* < 0.05) was significantly lower in sh-G*LI1* cells than in the WC and negative control groups. The mRNA expression of *MMP14* (*P* < 0.01), *MMP2* (*P* < 0.01) and *LAMC2* (*P* < 0.01) was significantly higher in OE-*GLI1* cells than the WC and negative control groups (Figure 9A and 9B). Western blotting confirmed that protein expression was consistent with that of the mRNAs (Figure 9C and 9D).

## DISCUSSION

VM occurs when highly malignant tumor cells interact with the extracellular matrix to form a vascular system that can transport blood, reshape the tumor microcirculation and connect with host blood vessels to obtain blood supply to tumors. In 1999, Maniotis et al. [18] discovered and defined VM in melanoma; since, such channels containing red blood cells but no endothelial cells, have been identified in a variety of malignant tumors [5, 8, 12, 19–21]. In recent years, it has been demonstrated that VM also exists in GC tissues, that it is closely associated with invasion and metastasis, and that VM is indicative of poor prognosis for GC patients [13, 22, 23]. At present, antiangiogenic targeted drug therapy is widely regarded as a rescue treatment for advanced GC [24–26]. However, the inhibitory effect on mimetic vessels independent of vascular endothelial cells is poor [22, 27]. Therefore, VM should be considered as a target for anti-GC therapy. In a previous study [13, 15], we observed that VM formation was positively associated with the expression of GAL-1 in primary GC tissue, and that GAL-1 can promote VM formation in GC through upregulating EMT signaling. However, the mechanism by which GAL-1 promotes VM via EMT in GC remain unknown. Numerous signaling pathways participate in the regulation of EMT through cooperation and antagonism, such as Ras-MAPK, Rho, Src-FAK, ERK1/2, PI3K/AKT and Hh [28–31]. GAL-1 can activate the Hh signaling pathway and promotes EMT in GC cells [32]. However, whether GAL-1 induces VM through Hh-GLI signaling activation via EMT in GC requires further exploration.

The Hh/GLI signal pathway was first identified in Drosophila [33] and is controlled by two receptors on the target cell membrane: patched (Ptc) and smoothened (Smo). Ptc is composed of 12 transmembrane domains, that can bind directly to the ligand and negatively regulate Hh signaling. Smo is a G-protein coupled transmembrane protein and is an essential receptor for Hh signal transmission. The nuclear factors involved in Hh signal transduction include transcription factor Ci/GLI, protein kinase A (PKA), etc. In standard conditions, Ptc inhibits the activity of Smo protein, thereby inhibiting the downstream pathway. Canonical Hh signaling involves Ptc binding of Hh, relieving inhibition of Smo and prompting GLI proteins to enter the nucleus and activate transcription of downstream target genes [34].

During embryogenesis, Hh/GLI signaling is activated and regulates cell proliferation and differentiation, whereas it is silent in adults [35]. However, Hh/GLI hyperactivation is associated with carcinogenesis in a variety of human malignancies including GC [17, 35–37]. We used IHC to detect the GLI1 protein, a marker of Hh signaling in 127 cases of GC and matched non-tumor tissues. We found that GLI1 expression in GC tissues was significantly higher than that in matched non-cancerous tissues. This result is similar to that of a study in non-small cell lung cancer [38]. We also found that the GAL-1 IHC scores of GLI1-positive GC tissues were significantly higher than those of GLI1-negative GC tissues (GAL-1 IHC scores positively correlate with GLI1 IHC scores in GC tissues). Clearly, these results indicate that GAL-1 is closely associated with GLI1 in GC.

Hh/GLI1 signaling is a promising target for cancer therapies, as the pathway controls major biological hallmarks of cancer, including proliferation, metastasis, survival, self-renewal and angiogenesis [39, 40]. Our previous study demonstrated that GAL-1 can induce EMT through upregulation of the Hh signaling pathway via GLI1 in GC [16]. We have also previously verified that cancer-associated fibroblasts induce EMT through β1 integrin-mediated upregulation of GLI1 in GC [17]. The present study demonstrates a positive correlation between GAL-1 and GLI1 expression in GC tissues, and further work *in vitro* and *in vivo* confirms that GAL-1/*LGALS1* promotes the expression of GLI1. These findings suggest that GAL-1 may activate the Hh/GLI1 signaling pathway in GC. Intracellular GAL-1 acts as a scaffold protein for intracellular signaling pathways in a carbohydrate-independent manner. Intracellular GAL-1 is a major regulator of H-Ras nanoclusters [41–43], and Ras signaling is known to induce or enhance Sonic Hedgehog (SHh) expression [44]. We speculate that GAL-1/*LGALS1* activates the Hh/GLI1 signaling pathway via Ras/SHh.

Although numerous studies have confirmed that the Hh/GLI1 pathway promotes tumor invasion and metastasis, the role of Hh/GLI signaling in VM has not been previously studied. The findings of the present study suggest that GAL-1/*LGALS1* may activate the Hh/GLI1 signaling pathway in GC tissues. Three-dimensional culture showed that overexpression of GLI1 promotes VM *in vitro. In vivo* study also revealed that overexpression of *GLI1* can significantly promote VM and that *GLI1*-silencing can inhibit VM in subcutaneously implanted GC. To confirmed that GAL-1/*LGALS1* promotes the formation of VM in GC through promoting the expression of GLI1, we used GANT61, a GLI1-specific antagonist, to investigate whether GAL-1/*LGALS1* promotes VM in GC through activation of the Hh pathway. GANT61 effectively inhibited the formation of VM structures by MGC-803 and AGS cells. Our previous study found that silencing of *LGALS1* inhibit the VM structure formation ability of MGC-803 cells [15]. Interestingly, simultaneous silencing of *LGALS1* and overexpression of *GLI1* in MGC-803 cells and AGS cells rescued this inhibition in tube-formation capacity. This indicates that *GLI1* effects on VM formation independent of *LGALS1.*

It has been reported that MMPs-Ln5γ2 is the final step of the VM signaling pathway [45]. In this study, we confirmed that *GLI1* promotes the expression of *MMP14*, *MMP2* and *LAMC2* mRNA and protein in MGC-803 and AGS cells. These results support the hypothesis that GAL-1 promotes the initiation of VM through Hh/GLI signaling in GC.

## CONCLUSIONS

Taken together, the results of this study suggest that Hh/GLI signaling pathway components are activated in GC by GAL-1/*LGALS1*, and that GLI1 controls initiation of VM in GC. These findings provide evidence that the GAL-1/Hh/GLI1 pathway plays a role in VM in GC. This provides novel insight into the mechanisms underlying VM, and may contribute towards the identification of a therapeutic target for GC. However, further study is required to understand the mechanism of GAL-1/*LGALS1* activation of GLI1, GLI1 transcriptional modification and the mechanism of VM initiation. Inhibition of these mechanisms may have the potential to overcome VM in GC and improve therapeutic outcomes for patients with refractory GC.

## MATERIALS AND METHODS

### Tumor tissue samples

We enrolled 127 patients with gastric adenocarcinoma. No patients had received preoperative neoadjuvant chemotherapy or radiotherapy, but all patients underwent radical gastrectomy and D2 lymphadenectomy at the Gastrointestinal Surgery Department, Taizhou People’s Hospital of Jiangsu province. GC tissue and matching adjacent non-tumor tissue for immunohistochemistry (IHC) and histochemical staining were formalin-fixed and paraffin-embedded. GC tissue and matching adjacent non-tumor tissue was collected from 15 patients for molecular analysis. Our study was approved by the Clinical Research Ethics Committee of Taizhou People’s Hospital (TZRY-EC-12-068).

### Animal models

Five-week-old male athymic mice were purchased from the Comparative Medicine Centre of Yangzhou University (Yang Zhou, JiangSu, China), and used to establish subcutaneous GC implantation. These experiments were approved by the Ethics Committee of Yang Zhou University (YZU-EC-JS2352). The mice were bred in a laminar flow cabinet under pathogen-free conditions. *LGALS1*-overexpressing (OE-*LGALS1*) MGC-803 cells, *LGALS1-* silencing (sh-*LGALS1*) MGC-803 cells, *GLI1*-overexpressing (OE-*GLI1*) MGC-803 cells, silencing *GLI1* MGC-803 cells (sh-*GLI1*), wild-type control MGC-803 cells or vector control MGC-803 cells were inoculated into the right flank (2x10^6^ cells/mouse; n = 6). Mice were sacrificed on day 21 and the subcutaneous GC tumors were harvested. Subcutaneous GC tumors were examined by hematoxylin and eosin (H&E) staining and CD34-PAS dual staining.

### Reagents and antibodies

GANT61 was purchased from Sigma Biotechnology (St. Louis, MO, USA). The anti- GAL-1 antibody was purchased from Cell Signaling Technology (13888S and 12936S, Danvers, MA, USA), the anti-GLI1 was purchased from Abcam (ab217326, Cambridge, UK) and Bioss (bs-1206R, Beijing, China), anti-MMP14 (ab78738), anti-MMP2(ab92536), anti-LAMC2 (ab210959 and ab274376) and anti-CD34(ab81289) antibodies were purchased from Abcam (Cambridge, UK), and the anti-GAPDH antibody(sc-47724), horseradish peroxidase (HRP)-conjugated goat anti-mouse IgG(sc-516102) and goat anti-rabbit IgG(sc-2357) were provided by Santa Cruz Biotechnology (Santa Cruz, CA, USA). The PAS staining kit was provided by Leagene Biotechnology Co, Ltd (Beijing, People’s Republic of China). The MTT assay kit and dimethyl sulfoxide (DMSO) were provided by Sigma Biotechnology (St. Louis, MO, USA).

### Cell line and cell culture

The human gastric adenocarcinoma cell line, MGC-803, was provided by The Type Culture Collection of the Chinese Academy of Sciences (Shanghai, China). Cells were cultured in RPMI (Thermo Fisher Scientific, Waltham, MA, USA) supplemented with 10% (V/V) fetal bovine serum (FBS; Thermo Fisher Scientific), 100 U/ml penicillin and 100 mg/ml streptomycin (Gibco, Grand Island, Waltham, MA, USA). Cells were maintained at 37°C in a humidified atmosphere containing 5% (V/V) CO_2_ [15], and passaged via trypsinization when 80% confluence was reached.

### Lentiviral transduction

Lentiviral transduction was performed as previously described [14]. Lentiviral vectors for *LGALS1* and *GLI1* overexpression and silencing were constructed by Genechem Co. Ltd (Shanghai, China). Three different short hair RNAs (shRNAs) were designed against both *LGALS1* and *GLI1* and cloned into the GV248(hU6-MCS-Ubiquitin-EGFP-IRES-puromycin) vector. The shRNA sequences of for *LGALS1* and *GLI1* are shown in Table 1. The GV358 (Ubi-MCS-3FLAG-SV40-EGFP-IRES-puromycin) lentiviral vector was constructed to upregulate the expression of *LGALS1* and *GLI1*. Before lentiviral transduction, MGC-803 or AGS cells were seeded in 6-well plates at a concentration of 5x10^4^ cells per well. According to a multiplicity of infection of 10, cells were transduced with lentiviral vector and 10 μg/ml polybrene (Sigma-Aldrich, St. Louis, MO, USA). Additionally, a non-targeting negative control lentiviral vector was transduced by the same approach. Medium was replaced 12 h after transduction, and 2 μg/ml puromycin (Sigma-Aldrich) was added after a further 48 h for selection of stably transduced cell lines. The stably transduced cells were cultured at 37°C in the presence of 0.5 μg/mL puromycin. After 72 h, transduction efficiency was evaluated using a fluorescent microscope (OLYMPUS-U-HGLGPS-IX73), and further confirmed by quantitative reverse transcription-PCR (qRT-PCR) or western blotting.

**Table 1:** shRNA sequences for knockdown of *GLI1* and *LGALS1.*

**Name**	**5’**	**Stem**	**Loop**	**Stem**	**3’**
*GLI1*-shRNA1	CCGG	GCCACCAAGCTAACCTCATGT	CTCGAG	GTTTCATACACAGATTCAGGC	TTTTTTG
*GLI1*-shRNA2	CCGG	GCCTGAATCTGTGTATGAAAC	CTCGAG	GTTTCATACACAGATTCAGGC	TTTTTTG
*GLI1*-shRNA3	CCGG	GCAGTAAAGCCTTCAGCAATG	CTCGAG	CATTGCTGAAGGCTTTACTGC	TTTTTTG
*LGALS1*-shRNA1	CCGG	GCTGCCAGATGGATACGAATT	CTCGAG	AATTCGTATCCATCTGGCAGC	TTTTTG
*LGALS1*-shRNA2	CCGG	CCAGCCTGGAAGTGTTGCAGA	CTCGAG	TCTGCAACACTTCCAGGCTGG	TTTTTG
*LGALS1*-shRNA3	CCGG	CACCATCGTGTGCAACAGCAA	CTCGAG	TTGCTGTTGCACACGATGGTG	TTTTTG
Control-shRNA	CCGG	TTCTCCGAACGTGTCACGT	TTCAAGAGA	ACGTGACACGTTCGGAGAA	TTTTTG

### RNA extraction and qRT-PCR

Total RNA was extracted using an RNeasy Mini Kit (Invitrogen, Waltham, MA, USA), and cDNA was synthesized using a Reverse transcription kit (Takara, Shiga, Japan), according to manufacturer’s instructions. qRT-PCR was performed with SYBR Green dye (Roche Diagnostics, Mannheim, Germany) and analyzed using an iQ5 Multicolor Real-Time PCR Detection System (Bio-Rad, Hercules, CA, USA). The thermocycling conditions were as follows: 95°C for 30 s, then by 40 cycles of 95°C for 5 s, 60°C for 30 s and 72°C for 30 s. Glyceraldehyde phosphate dehydrogenase (GAPDH) was used as the reference control gene. The primers sequences used are listed in Table 2.

**Table 2:** Primer sequences used for quantitative RT-PCR.

**Gene**	**Primer sequence (5’ to 3’)**
*GLI1*	F: GGGATGATCCCACATCCTCAGTC
	R: CTGGAGCAGCCCCCCCAGT
*LGALS1*	F: GCTGAACCTGGGCAAAGACAG
	R: GTTGAGGCGGTTGGGGAACTT
*MMP-14*	F: CTGCGTCCATCAACACTGCCTA
	R: GCCCAGCTCCTTAATGTGCTTG
*MMP-2*	F: GGCGGTCACAGCTACTTCTTC
	R: GCAGCCTAGCCAGTCGGATT
*LAMC2*	F: TCGGGAGCCATGTCATGTGAGTG
	R: CCCAGCATCAGGAAGCAAGGAGT
*GAPDH*	F: TGACTTCAACAGCGACACCCA
	R: CACCCTGTTGCTGTAGCCAAA

### Western blotting

Extraction kit (Beyotime, Shanghai, China) was used to isolate total-cell extracts and nuclear extracts. For all samples, 20 μg cell lysate was separated by 12% sodium dodecyl sulfate-polyacrylamide gel electrophoresis (SDS-PAGE) and the separated proteins were transferred into a nitrocellulose membrane (GE Healthcare Life Sciences, Pittsburgh, PA, USA). Blots were probed with anti-GLI1, anti-GAL-1, anti-MMP14, anti-MMP2, anti-LAMC2 or anti-GAPDH primary antibody at a dilution of 1:2,000. The secondary HRP antibody was also used at a dilution of 1:2,000. Protein bands were visualized using West Picochemiluminescent Substrate (Pierce, Carlsbad, CA, USA) and quantified using densitometric image analysis software (Image Master VDS; Pharmacia Biotech). GAPDH was used as a loading control. All western blotting analysis assays were performed in triplicate.

### Cell viability assay

The 3-(4-5-dimethylthiazol-2-yl)-2, 5-diphenyltetrazoliumbromide dye reduction assay (MTT assay) was used to determine cell viability. Cells were seeded into a 96-well flat bottom plate at 5×10^3^ cells/well. The cells were cultured at 37°C with 5% CO_2_ in a humidified atmosphere. After an overnight incubation, the cells were treated with various concentrations of GANT61 (5μM, 10 μM, 15 μM and 20 μM). After GANT61 treatment for 24 h, 20 μL MTT was added to each well and incubated for 4 h. Following the subsequent removal of the supernatant, 150 μL dimethyl sulfoxide (DMSO) was added to dissolve the formazan crystals. Absorbance was measured at 490 nm with an Enzyme-linked Immunosorbent Detector (Model 550; Bio-Rad, Hercules, CA, USA).

### IHC

IHC was performed for human GC tissues, matching adjacent non-tumor tissue and murine subcutaneous GC tumor tissue. GAL-1 (dilution, 1:200), GLI1 (dilution, 1:200) or CD34 (dilution, 1:100) primary antibodies were incubated with the slides overnight at 4°C, the following steps were performed as per our previous report [13]. GAL-1 and GLI1 staining was imaged digitally using the same light exposure and evaluated using Image Pro Plus (IPP), a digitalized IHC scoring program (Media Cybernetics, San Diego, CA). The immunostaining results are expressed as the percentage of positively stained cells multiplied by staining intensity score (0, 1, 2 or 3) to yield scores of 0-300.

### Three-dimensional culture

Coating of 24-well plates with 200 μl growth factor-reduced Matrigel (BD Biosciences, San Diego, CA, USA) was carried out, and incubated at 37°C for 1 h for polymerization. A total of 1x10^5^ cells in 600 μl of medium were plated onto the polymerized gel and incubated cells at 37°C for 24 h. The cells were imaged under an inverted microscope (OLYMPUS-U-HGLGPS-IX73). All experiments were performed in triplicate.

### CD34-PAS dual staining and VM evaluation

All specimens were formalin-fixed and paraffin-embedded. After CD34 IHC described above, PAS staining was performed, according to the kit manufacturer’s protocol. After the DAB reaction, sections were treated with 0.5% periodic acid solution for 10 min. Prior to staining with Schiff solution for 20 min at room temperature, sections were washed with distilled water 3 times. Sections were then counterstained with hematoxylin, dehydrated, cleared and mounted. VM was analyzed at x200 magnification in 10 randomly selected fields of view. Channel-like structures stained positively for PAS and negatively for CD34, and containing red cells were considered to have a VM-positive status.

### Statistical analysis

Statistical analysis was conducted using SPSS software (version 16; Chicago, IL, USA). Categorical variables were compared using the Chi-squared test. Continuous variables are presented as the mean ± standard error of the mean, Student’s t-test was used to compare between groups based on the normal distribution of the data. Pearson or Spearman’s correlation coefficient were used to determine the relationship between two variables. *P* < 0.05 was considered to indicate a statistically significant difference.

### Ethical approval

This clinical study was approved by the Clinical Research Ethics Committee of Taizhou People’s Hospital (TZRY-EC-12-068), all patients consented to participate in our study. The animal experiments were approved by the Ethics Committee of Yang Zhou University (YZU-EC-JS2352).
